# Prolonged neuromuscular block in a preeclamptic patient induced by magnesium sulfate

**DOI:** 10.11604/pamj.2016.25.5.6616

**Published:** 2016-09-14

**Authors:** Mohamed Adnane Berdai, Smael Labib, Mustapha Harandou

**Affiliations:** 1 Obstetric and Pediatric Intensive Care Unit, University Hospital Hassan II, Fez, Morocco

**Keywords:** Magnesium sulfate, non depolarizing relaxants, pregnancy, neuromuscular block

## Abstract

Recent large use of magnesium in the obstetric population should incite anesthesiologists to control its side effects and drugs interactions. We report a case of a 30-year-old woman, with severe preeclampsia and HELLP syndrome, receiving sulfate magnesium and nicardipine, who underwent a cesarean section under general anesthesia. She developed a prolonged and deep neuromuscular blockade, which was antagonized three hours later with neostigmine. In case of therapeutic hypermagnesaemia, non-depolarizing relaxants must be used in reduced doses, and at increased time intervals, with appropriate neuromuscular monitoring.

## Introduction

Magnesium sulfate is widely used in preeclampsia patients to prevent and to treat seizures. It is also used as a tocolytic agent and for labour analgesia. However, its potentiating effects on non depolarizing neuromuscular blocking agents should be taken in consideration, especially in obstetric patients. We report the case of prolonged neuromuscular blockade after cesarean section in a patient receiving magnesium and nicardipine.

## Patient and observation

A 30-year-old woman, primigravida, at 33 weeks of pregnancy, with unremarkable medical history. She presented to the hospital with complains of headache and epigastric pain. The patient weighted 60 kg, was conscious, her blood pressure was 160/110 mmHg, pulse of 120/min, and respiratory rate of 16/min, no edema was present. Blood cell count revealed a hemoglobin level of 11 g/dl, platelet count of 31000 cells/ml, coagulation test and kidney function tests were normal, liver function tests showed L-aspartate aminotransferase level at 231 IU/l (N<40), L-alanine aminotransferase at 114 IU/l (N<45), proteinuria was mild (600 mg/24h). We concluded to the diagnosis of pregnancy induced severe preeclampsia associated with incomplete HELLP syndrome. An intravenous infusion of magnesium sulfate was given with a 4 g loading dose perfused in 30 minutes and continued at 1 g/hour. Nicardipine was selected as the antihypertensive drug (2 mg/h). Due to continuing symptoms of headache and epigastric pain, elevated blood pressure (175/123 mmHg), and severe thrombocytopenia; decision of emergency delivery was made and was undertaken by caesarean section. Regional anesthesia wasn't appropriate because of severe thrombocytopenia, We performed then general anesthesia. Standard monitoring was used. After 3 minutes of preoxygenation, anesthesia was induced with propofol 150 mg and vecuronium 6 mg. Oral endotracheal tube was inserted without difficulty 100 seconds after muscle relaxant administration. Anesthesia was maintained with isoflurane (1-1.2 MAC), 50% nitrous oxide in oxygen, and fentanyl 150 µg after delivery of the baby. At this time, she received 35 mg/kg of amoxicillin plus clavulanic acid as antibioprophylaxy. The end-tidal CO2 was maintained at 36-38 mmHg. During surgery, blood loss was estimated at 800 ml, we transfused one unit of packed red blood cells. The cesarean section ended uneventfully at 40 min postinduction. The patient received then magnesium sulfate 1g/h and nicardipine 1 mg/h. Due to the context of severe preeclampsia and HELLP syndrome, the patient was shifted to the intensive care unit of the obstetric department for close observation and extubation. At admission, she was unconscient, with blood pressure at 150/110 mmHg and pulse of 128/min, the urine output was 2.1 ml/kg/j. One hour after the end of surgery, we noted the absence of awakening signs. In order to search neurological complication of preeclampsia, we realized a cerebral tomodensitometry which was normal. Three hours later, we suspected a prolonged neuromuscular blockade, so, we administrated 40 µg/kg of neostigmine and 20 µg/kg of atropine. Five minutes later, we noted the reappearance of coughing and swallowing reflexes, and in 10 minutes, the patient executed verbal orders. Therefore, she was successfully extubated without any residual neuromuscular blockade. Liver and hematologic biological abnormalities were progressively corrected. On the third postoperative day, the patient was discharged.

**Consent:** Written informed consent was obtained from the patient for publication of this Case report and any accompanying images. A copy of the written consent is available for review by the Editor-in-Chief of this journal.

## Discussion

Magnesium sulfate is nowadays established as the treatment of choice for the prevention and control of eclamptic convulsions [1], It can also be used at caesarean delivery to attenuate hypertensive response to tracheal intubation, and can be indicated for fetal neuroprotection in preterm labour, and as a tocolytic agent [2]. Magnesium can also be administrated as an adjunct for labour analgesia or postoperative pain relief, its administration reduces postoperative pain and opioid consumption [3]. With this recent large use of magnesium in the obstetric population, a parturient has a greater likelihood of hypermagnesaemia. In this situation, clinicians should take in consideration the interaction of this ion with muscles relaxants. In fact, magnesium suppresses acetylcholine transmission by blocking the calcium channel at the motor endplate, and by decreasing pre-synaptic release of acetylcholine [2]. Magnesium alone can produce neuromuscular blockade at serum levels above 5 mmol/l, hence the use of loss of patellar reflexes as a clinical monitor for magnesium toxicity [4]. As result of diminishing muscle fiber excitability and reducing the amplitude of endplate potential, magnesium potentiates induced neuromuscular blockade by nondepolarizing neuromuscular blocker [5]. In our case, neuromuscular monitoring wasn't available, and due to pre-eclampsia context, we suspected initially a neurological complication. The diagnosis was rectified later by suspecting an interaction of magnesium and vecuronium which would result in such severe prolongation of neuromuscular blockade. This diagnosis of prolonged neuromuscular blockade was based on its reversibility by using anti-cholinesterase. Several case reports have highlighted the potential for clinical difficulties with non-depolarizing muscle relaxants in obstetric patients with therapeutic hypermagnesaemia. Yoshida et al. reported prolonged ventilatory support in two parturients receiving vecuronium following administration of magnesium for preeclampsia and tocolysis [6]. The magnesium potentiates the action of all non-depolarizing neuromuscular blockers; the speed of onset of pancuronium and atracurium are increased by administration of magnesium, and the duration of the neuromuscular block induced by rocuronium is prolonged [7]. The onset and duration of neuromuscular blockade produced by suxamethonium is not potentiated in hypermagnesemic states. However, magnesium may reduce the fasciculations seen on induction of the neuromuscular block [2]. In our case, we used vecuronium as a myorelaxant for intubation and maintaining neuromuscular blockade during cesarean section, because it was the only disponible myorelaxant agent. Fuchs-Buder et al. demonstrate that magnesium, administered before vecuronium, accelerated the onset, intensified and prolonged neuromuscular block, and concluded that monitoring of neuromuscular function and reduction in dose of vecuronium are required when using these two drugs in combination [8]. There are case reports describing maternal neuromuscular blockade in concomitant administration of magnesium and calcium channel blockers. Even if, this interaction in pregnant woman is rare and not formally proved, it may be a possible [9], we believe that neuromuscular monitoring in this context is indicated. In patients who had received intravenous magnesium, the speed of recovery after antagonizing a vecuronium-induced neuromuscular blockade with neostigmine is decreased by approximately 30%, thus, this attenuation is mainly a result of slower spontaneous recovery and not decreasing response to neostigmine [10]. Unlike cholinesterase inhibitors, sugammadex encapsulates steroidal muscle relaxants. It causes a rapid and complete reversal of the neuromuscular blockade. Administration of magnesium before induction of anesthesia had no effect on the ability of recommended doses of sugammadex to reverse neuromuscular blockade [11].

## Conclusion

Where relaxants are to be used in a magnesium treated patient with obstetric condition, non-depolarizing agents must be used in reduced doses, and at increased time intervals. The neuromuscular monitoring in this context is essential. Concomitant administration of calcium channel blockers can also potentiate neuromuscular blockade, caution is advised when these agents are combined.
